# Quantitative Oculomotor and Vestibular Profile in Spinocerebellar Ataxia Type 6 – Systematic Review and Meta-Analysis

**DOI:** 10.1007/s12311-024-01774-y

**Published:** 2024-12-15

**Authors:** Alexander A. Tarnutzer, Pilar Garces, Chrystalina A. Antoniades

**Affiliations:** 1 Cantonal Hospital of Baden, Baden, Switzerland; 2 Faculty of Medicine, University of Zurich, Zurich, Switzerland; 3 Roche Pharma Research and Early Development, Neuroscience and Rare Diseases, Roche Innovation Center Basel, Basel, Switzerland; 4 NeuroMetrology Lab, Nuffield Department of Clinical Neurosciences, Clinical Neurology, Medical Sciences Division, University of Oxford, Oxford, OX3 9DU UK

**Keywords:** Oculomotor, Vestibular, Eye movement recordings, Hereditary ataxia, Systematic review, Recommendations

## Abstract

Whereas several studies have reported on quantitative oculomotor and vestibular measurements in spinocerebellar ataxia type 6 (SCA6), selecting the most suitable paradigms remains challenging. We aimed to address this knowledge gap through a systematic literature review and providing disease-specific recommendations for a tailored set of eye-movement recordings in SCA6. A literature search (MEDLINE, Embase) was performed focusing on studies reporting on quantitative oculomotor and/or vestibular measurements in SCA6-patients. Oculomotor and vestibular parameters were extracted and correlations with various epidemiologic and clinical parameters were sought. Twenty-two studies were included reporting on 154 patients. Abnormalities observed included reduced pursuit gain (58/69), frequent square-wave jerks (23/40), spontaneous downbeat nystagmus (DBN, 34/55) and triggered nystagmus including positional nystagmus (25/34) and vertical (“perverted”) head-shaking nystagmus (21/34), gaze-evoked nystagmus (48/70) and angular vestibulo-ocular reflex (aVOR)-suppression (21/25), and high-frequency aVOR-deficits (26/33). For horizontal visually-guided saccades (VGS), changes in metrics (36/66) were frequently observed, whereas saccade velocity was usually preserved (39/44) and saccade latency within normal limits. Reduced high-frequency aVOR gains, VGS-latency and metrics correlated with disease severity. Longitudinal data indicated deterioration of individual video-head-impulse testing gains over time. A broad range of oculomotor and vestibular domains are affected in SCA6. Impairments in pursuit, saccade metrics, gaze-holding (gaze-evoked nystagmus, DBN) and high-frequency aVOR were most frequently identified and as such, should be prioritized as disease markers. Quantitative oculomotor testing in SCA6 may facilitate an early diagnosis and prove valuable in monitoring disease progression.

**Supplementary Information:**

The online version contains supplementary material available at 10.1007/s12311-024-01774-y.

## Introduction

Oculomotor abnormalities are frequently observed in spinocerebellar ataxia type 6 (SCA6) [1], which is an adult-onset, autosomal dominant hereditary cerebellar ataxia (CA). It is caused by pathologically expanded CAG trinucleotide repeats (range = 20–33 repeats) in the CACNA1A gene, which encodes for the α−1A subunit of the P/Q type voltage-gated calcium channel [2, 3]. The average age of symptom onset for SCA6 typically ranges from 42 to 53 years, with an overall range of 19 to 73 years [2]. The primary clinical features include gradually worsening cerebellar ataxia, dysarthria, and oculomotor impairments.

Eye movement abnormalities observed at the bedside have proven to be a sensitive and early marker of disease in CA [4]. Oculomotor abnormalities identified in SCA6 include impaired eccentric gaze holding resulting in gaze-evoked nystagmus (GEN) and downbeat nystagmus (DBN) [5] and are a critical factor in defining the clinical presentation of hereditary ataxias [6]. Pyramidal tract involvement may be seen in up to 50% of cases and extrapyramidal (basal ganglia) involvement (such as dystonia and blepharospasm) may be observed in up to 25%. Predominantly midline, paramedian vermian cerebellar atrophy due to loss of Purkinje cells may be seen on sagittal MR-imaging, being in line with neuropathological studies indicating pure cerebellar atrophy in SCA6, with other structures such as the brainstem being rarely affected [7]. Eye movement abnormalities may be the only motor symptom in early or pre-manifest stages of the disease, making their evaluation essential for accurate diagnosis and importantly for differential diagnosis. In comparison to other motor findings, eye movements have the advantage that they are relatively well studied and not confounded by significant inertia or musculoskeletal factors [8]. With quantitative oculomotor and vestibular assessments being increasingly available, this allows for improved comparability over time, reduced examiner dependency, and increased sensitivity to subtle changes.

The primary goal of this study was to systematically review the literature on quantitative oculomotor and vestibular measurements in SCA6 patients and to identify the disease-specific pattern of eye-movement abnormalities both in ataxic patients and pre-symptomatic carriers. This will allow us to propose a core-set of oculomotor / vestibular paradigms (“digital oculomotor biomarkers”) that permits the detection of the most prominent and most frequently observed eye movement abnormalities in SCA6 [9]. Furthermore, the identification of distinguishing features from other hereditary ataxias was also an important goal, including the identification of gaps of knowledge to guide future research projects in the field.

## Material and Methods

### Data Sources and Searches

We searched MEDLINE (via PubMed) for articles using text words and controlled-vocabulary terms related to research studies reporting on oculomotor and/or vestibular function in hereditary ataxia. A detailed description of the search strategy can be found in Appendix 1. Our search was updated through May 7th 2024 and was identical to the search string previously used by our group [6, 8].

### Study Selection and Quality Rating

**Fig. 1:**
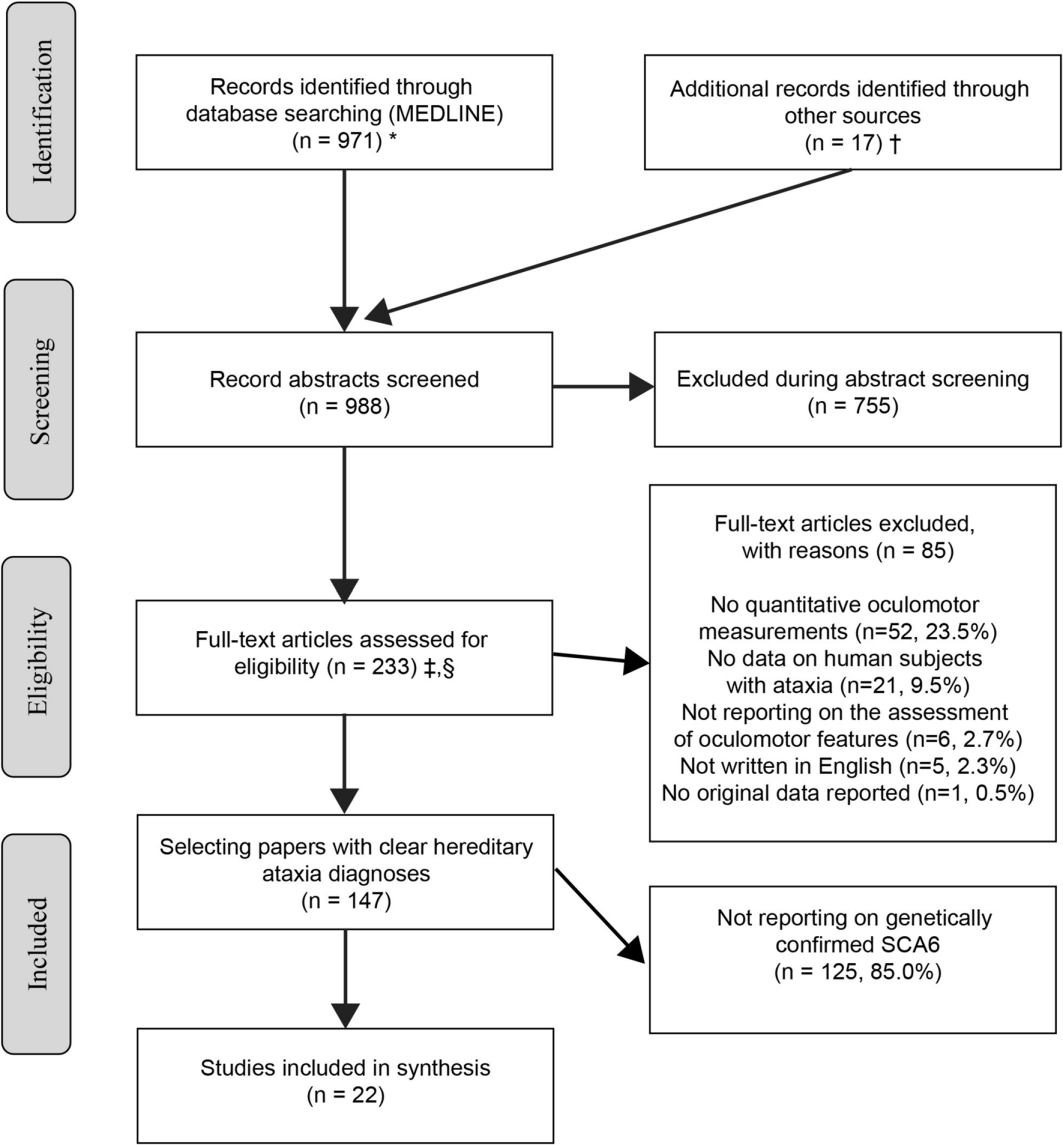
*MEDLINE was accessed via PubMed. † Individual hand search of citation lists from selected studies and investigator files identified 17 additional manuscripts for review. ‡ Abstracts coded as “yes” or “maybe” by at least one reviewer were included in full-text review. § After full-text evaluation by two reviewers, any differences were resolved by discussion and – if needed—adjudication by a third, independent reviewer

Articles were selected by two independent raters (PG and AAT) using pre-determined inclusion criteria and a structured protocol (see Appendix 1). For this review we included only English-language articles with original data on human subjects with genetically confirmed hereditary ataxia, as illustrated in the PRISMA flow-chart (Fig. 1). Our focus was on studies reporting on *quantitative* oculomotor and / or vestibular testing in *hereditary* ataxia, specifically in SCA6. We calculated inter-rater agreement on full-text inclusion using Cohen’s kappa.

A quality rating of included studies was performed based on eight predefined quality criteria covering items related to (i) study-cohort, (ii) data acquisition and (iii) data analysis (see Appendix 2 and our previous publication [6, 8]). An overall study quality rating (high, moderate, low) was derived from this quality assessment.

### Data Extraction, Data Synthesis and Statistical Analysis

From all eligible articles included in this meta-analysis, we extracted information regarding the study type and its sample size, the oculomotor paradigms applied and the eye movement recording device(s) used. Details about oculomotor and/or vestibular parameters reported, including pursuit eye movements (PEM), saccadic eye movements (SEM), fixation (including spontaneous nystagmus (SN) and eccentric gaze-holding (looking for gaze-evoked nystagmus (GEN)), saccadic intrusions (SI), triggered nystagmus (including positional nystagmus (PN), head-shaking nystagmus (HSN) and optokinetic nystagmus (OKN)) and the angular vestibulo-ocular reflex (aVOR) as measured with the quantitative head-impulse test (qHIT) or rotatory chair testing), were actively searched for and stored. Subsequently, we determined the frequency of presentation and the degree of abnormality for each oculomotor/vestibular parameter identified. For the frequency fraction calculation, only studies with frequency values or single patients’ values were considered, whereas for the degree of abnormality determination, studies with single patients’ values or mean values were contemplated. The frequency of presentation was then graded by symbols (from “- “ to “ +  +  + ”), each corresponding to a specific percentage range of presentation as previously done by our group [10]. Regarding the level of abnormality, the difference between the mean value of the patient group and that of the control group was calculated. The variation in percentage was graded from “↑↑↑” to “↓↓↓”, similarly to the frequency fractions. Where possible, a meta-analysis was performed. For most publications considered, this was not possible due to differing methodologies in oculomotor measurement paradigms.

We also searched for correlations between oculomotor parameters and established measures of disease severity such as clinical parameters (clinical scales including the Scale for the Assessment and Rating of Ataxia (SARA) [11] and the ICARS (International Cooperative Ataxia Rating Scale) [12], age, disease duration, age at onset), biological determinants (e.g., CAG repeat length), and/or (MR) imaging markers. This study is reported in accordance with PRISMA guidelines. [13]. Correlations with anchor measures were classified for studies reporting Pearson correlation coefficients or Spearman correlation coefficients (strong: *r* ≥ 0.7; moderate: 0.4 $\le$ *r* < 0.7; weak: 0.1 $\le$ *r* < 0.4) [14].

## Results

Our search identified 988 citations, of which 755 (76.4%) were excluded at the abstract level and 86 (8.7%) at the full-text manuscript level. Out of the 147 studies included after the full-text review, 22 studies (representing 2.2% of all manuscripts reviewing) reported on a total of 154 patients with genetically confirmed SCA6 (see PRISMA flow chart—Fig. 1 for details). Only one out of 22 studies reported on pre-symptomatic carriers (*n* = 4 patients) in SCA6 [15].

The overall study quality with respect to the predefined criteria (see Appendix 2) was judged ‘high’ in 7 studies (31.8%), ‘moderate’ in 11 studies (50.0%) and ‘low’ in 4 studies (18.2%) (see Appendix 3, Table S1 for details). The primary focus of the vast majority of studies was the phenotypic characterization of oculomotor abnormalities in the respective population (*n* = 18), whereas in another four studies the value of oculomotor parameters in the differential diagnosis was assessed. Twenty-one out of 22 studies were cross-sectional, a longitudinal study design (with a follow-up of up to five years) was used in a single study [16]. There were no SCA6 treatment trials identified reporting on quantitative oculomotor parameters.

### Setup, Paradigms and Quantitative Parameters

The studies included reported on a broad range of oculomotor paradigms that were obtained with a variety of recording techniques (see Table 1 and Appendix 3, Tables S2-S4 for details). Specifically, most frequently video-oculography (*n* = 9 studies, 72 patients) and scleral search-coil recordings (*n* = 6 studies, 40 patients) were applied, followed by electro-oculography (EOG) in 16 patients (*n* = 5 studies). Recordings were binocular in 35% of patients (49/139) and monocular in 46% of patients (64/139), whereas in the remaining 19% of patients (26/139) this information could not be retrieved. Eye movement recordings were obtained for both the horizontal and vertical plane in 69% of patients (96/139), whereas they were restricted to the horizontal plane in 31% of patients (43/139).

**Table 1:** Study designs and patient population

	Studies (*n*)	Patients (*n*)
Study location		
Monocentric	20 [15, 17–25, 27, 28, 42–49]	129
Multicentric	2 [16, 26]	25
Study type		
Case series	6 [16, 18, 19, 21, 26, 45]	40
Case control studies	16 [15, 17, 20, 22–25, 27, 28, 42–44, 46–49]	114
Study design		
Observational study prospective	1 [15]	9
Cross-sectional study, prospective	20 [17–28, 42–49]	133
Cross-sectional study, retrospective	1 [16]	12
Gender		
Female patients included	10 [15–17, 20–22, 25, 27, 28, 44]	42
Male patients included	12 [15–17, 20–22, 25, 27, 28, 43, 44, 46]	55
Gender not reported	10 [18, 19, 23, 24, 26, 42, 45, 47–49]	57
Patient cohort		
Pre-symptomatic carriers	1 [15]	4
Ataxic patients	22 [15–28, 42–49]	150
Overall study quality		
High	7 [17, 20, 23, 25, 28, 44, 47]	64
Moderate	11[15, 16, 18, 19, 21, 22, 24, 27, 46, 48, 49]	66
Low	4 [26, 42, 43, 45]	24
EM data collection – device(s) used		
Electro-oculography (EOG)	5 [18, 19, 21, 48, 49]	16
Electro-oculography or search coil	1 [28]	11
No EM data collected	1 [44]	15
Scleral search coils	6 [15, 17, 23, 24, 26, 43]	40
Video-oculography (VOG)	9 [16, 20, 22, 25, 27, 42, 45–47]	72
Plane of EM recording		
Horizontal plane only	11 [18–21, 23, 24, 42, 43, 45, 48, 49]	43
Horizontal and vertical plane	10 [15–17, 22, 25–28, 46, 47]	96
Number of eyes recorded		
Monocular recordings	9 [15, 17, 20, 24–26, 43, 46, 47]	64
Binocular recording	7 [16, 22, 23, 27, 42, 48, 49]	49
Not reported	5 [18, 19, 21, 28, 45]	26

Abbreviations:* EM* Eye movement, *SSC* scleral search coils, *VOG* video-oculography

Oculomotor paradigms most frequently obtained in SCA6 patients included visually-guided saccadic eye movements (VGS; *n* = 98 patients [14 studies]), pursuit eye movements (PEM; *n* = 72 patients [10 studies]), gaze holding at primary gaze position (*n* = 60 patients [7 studies]) and at eccentric gaze (*n* = 60 patients [7 studies]), and saccadic intrusions (*n* = 44 patients [5 studies]). Quantitative head-impulse testing (*n* = 33 patients [3 studies]) was more frequently applied than caloric irrigation (*n* = 14 patients [3 studies], but less often than rotational VOR testing (*n* = 40 patients [6 studies] (for details see Table 2 and Table S4 in appendix 3).

**Table 2:** Recorded oculomotor and vestibular parameters

	Studies (*n*)			Patients (*n*)		
	Horizontal plane only	Vertical and horizontal plane	Total	Horizontal plane only	Vertical and horizontal plane	Total
Gaze holding						
SN in primary gaze position	2 [18, 21]	5 [16, 17, 22, 26, 27]	7	8	52	60
Gaze-evoked nystagmus	2 [21, 26]	5 [16–18, 22, 27]	7	16	44	60
Rebound nystagmus	1 [21]	1 [18]	2	3	5	8
Triggered nystagmus						
Head-shaking nystagmus	-	3 [16, 22, 27]	3	0	34	34
Hyperventilation-induced nystagmus	-	1 [17]	1	0	5	5
Positional nystagmus	-	3 [16, 22, 27]	3	0	34	34
Pursuit eye movements	6 [18–21, 23, 42]	5 [15–17, 22, 26]	11	27	49	76
Saccadic eye movements						
Visually-guided saccades	8 [18, 19, 21, 24, 27, 45, 48, 49]	6 [15–17, 22, 25, 26]	14	37	61	98
Memory-guided saccades	2 [48, 49]	1 [25]	3	4	12	16
Saccadic intrusions	1 [18]	4 [15, 17, 26, 27]	5	5	39	44
VOR						
OKN	3 [18, 19, 21]	-	3	12	0	12
Caloric irrigation	2 [21, 28]	-	2	14	0	14
Decay TC	1 [26]	-	1	13	0	13
Rotational	7 [18–20, 23, 26, 28, 43]	-	7	44	0	44
Translational	2 [23, 43]	-	2	7	0	7
Vision-enhanced	2 [18, 20]	-	2	10	0	10
VOR high-frequency	-	3 [16, 22, 28]	3	0	33	33
VOR-suppression	5 [16, 18–20, 26]	-	5	39	0	39

Abbreviations:* EM* eye movement, *OKN* Optokinetic nystagmus, *PEM* pursuit eye movements, *SN* Spontaneous nystagmus, *TC* Time constant,*VOR* Vestibulo-ocular reflex

### Oculomotor Findings

#### Pursuit Eye Movements (PEM)

**Fig. 2:**
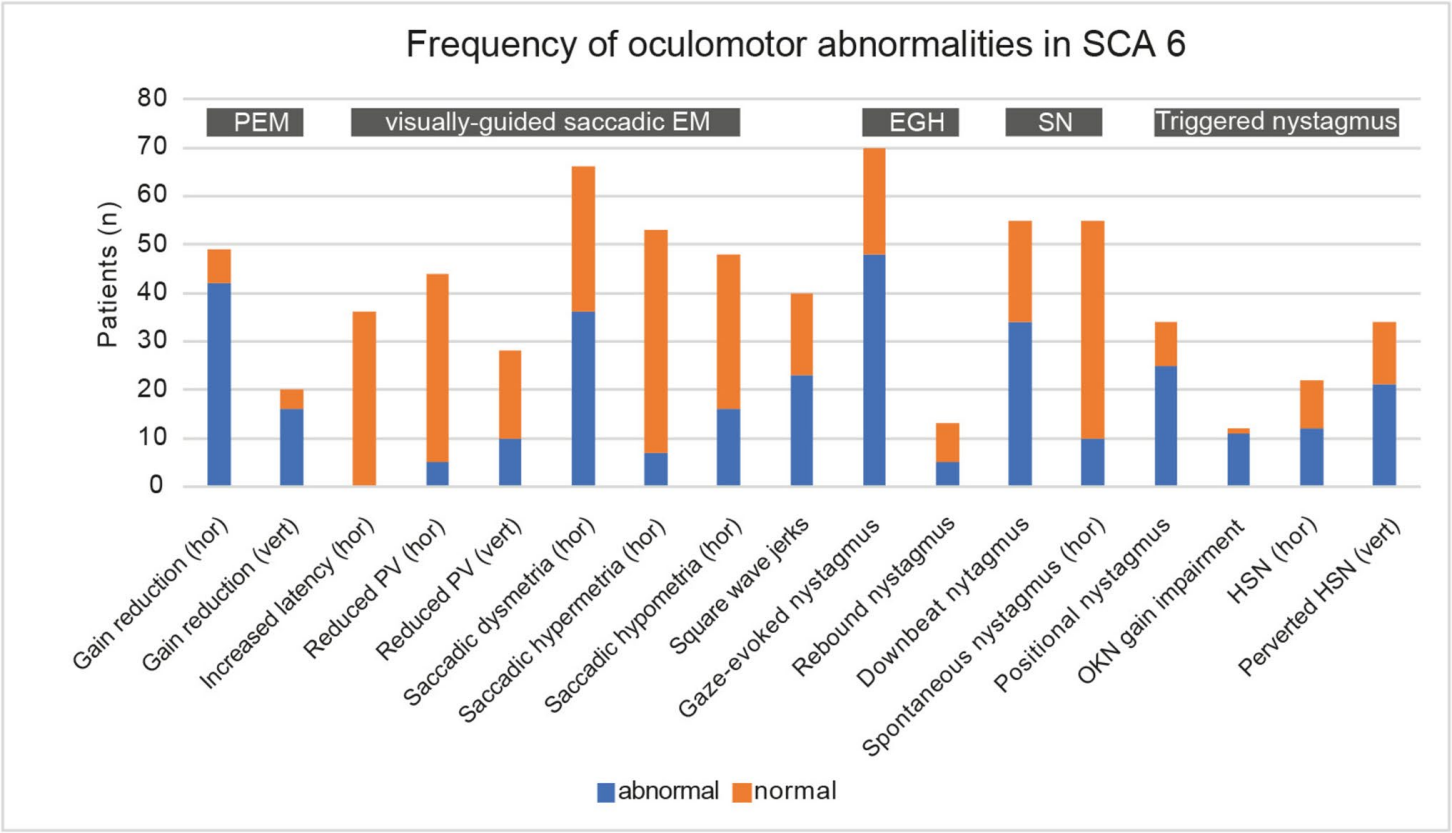
Frequency of oculomotor abnormalities amongst all SCA6 patients included. Thereby the number of SCA6-patients with abnormal (in blue) and normal (in red) findings for each oculomotor parameter is shown. Different oculomotor domains (pursuit eye movements, visually-guided saccadic eye movements, saccadic intrusions, eccentric gaze holding deficits, spontaneous and triggered nystagmus were grouped. Abbreviations: EGH = eccentric gaze holding; EM = eye movements; hor = horizontal; HSN = head-shaking nystagmus; OKN = optokinetic nystagmus; PEM = pursuit eye movements; PV = peak velocity; SCA6 = spinocerebellar ataxia type 6; SN = spontaneous nystagmus; vert = vertical

PEM gain was reduced in almost all patients across the eight studies that reported on this parameter (84%, 58/69) (see Figure 2 and Table 3). This was true both for horizontal PEM gains (86%, 42/49 patients from 8 studies [15–22]) and for vertical PEM gains (80%, 16/20 patients from 3 studies [15, 17, 22]). Severe PEM gain reductions were seen over a broad range of sinusoidal stimulus paradigms with distinct peak velocities (ranging from 12.3 to 45 deg/sec) and frequencies (0.2 Hz and 0.4 Hz). The mean gain-values for three studies employing identical recording paradigms (sinusoidal movements at 0.4 Hz, peak stimulus velocity of 45 deg/sec) ranged between 0.33 ± 0.11 and 0.41 ± 0.19, compared to healthy controls with values in the range of 0.88 ± 0.8 and 0.87 ± 0.11 [18, 19, 23]. Likewise, single studies demonstrated severe PEM gain reductions as well for lower frequency (0.2 Hz, peak velocity 22.6 deg/sec) sinusoidal stimuli (0.46 ± 0.06 vs. 0.92 ± 0.05, SCA6 patients vs. healthy controls) [19] and lower peak velocities (12.6 deg/sec) at 0.4 Hz sinusoidal stimulation (0.26 ± 0.06 vs. 0.91 ± 0.07) [20].

**Table 3:** Overview of oculomotor findings – level of abnormality and frequency of presentation

	Level of abnormality*	*n* (patients)	Frequency of presentation	*n* (patients)
Pursuit eye movements				
Horizontal pursuit gain	↓↓↓ [15–22]	49	+ + + [15–22]	49
Vertical pursuit gain	↓↓↓ [15, 17, 22]	20	+ + + [15, 17, 22]	20
Saccadic eye movements				
Horizontal VGS – increased latency	∅ [17, 18, 24, 25]	36	∅ [17, 18, 24, 25]	36
Horizontal VGS – decreased PV	∅—↓ [17–19, 22, 25, 26]	44	= / + [17–19, 22, 25, 26]	44
Vertical VGS – decreased PV	↓↓ [15, 17, 22, 26]	28	+ [15, 17, 22, 26]	28
Horizontal VGS—dysmetria	∅—↓/↑ [15, 17–19, 25]	31	+ + [15–19, 21, 22, 25, 27]	66
Horizontal VGS—hypermetria	∅—↑ [15, 17–19, 22, 25]	41	= / + [15–19, 22, 25]	53
Horizontal VGS—hypometria	∅—↓ [15, 17–19, 22, 25]	41	+ [15–17, 19, 22, 25]	48
Horizontal MGS—latency	∅ [25]	12	∅ [25]	12
Horizontal MGS—accuracy	∅ [25]	12	∅ [25]	12
Saccadic intrusions				
Square-wave jerks	NR	NR	+ + [15, 17, 18, 26, 27]	40
Spontaneous nystagmus				
Downbeat nystagmus	NR	NR	+ + [16, 17, 21, 22, 26, 27]	55
Horizontal nystagmus	NR	NR	= / + [16, 17, 21, 22, 26, 27]	55
Eccentric gaze holding				
Horizontal gaze-evoked nystagmus	NR	NR	+ + [16–18, 21, 22, 26, 27]	70
Rebound nystagmus	NR	NR	+ [17, 18, 21]	13
Triggered nystagmus				
Horizontal head-shaking nystagmus	NR	NR	+ + [22, 27]	22
Vertical (“perverted”) head-shaking nystagmus	NR	NR	+ + [16, 22, 27]	34
Positional nystagmus	NR	NR	+ + + [16, 22, 27]	34

Symbol legend:

Level of abnormality:

↑/ ↓ = reduced/increased by 5–25% (mildly)

↑↑/↓↓ = reduced/increased by 25–50% (moderately)

↑↑↑/↓↓↓ = reduced/increased by > 50% (strongly)

Frequency of presentation:

∅ = not present

= / +  = fraction 5–20% (rarely)

+  = fraction 20–40% (sometimes)

+  +  = fraction 40–70% (in a significant fraction)

+  +  +  = fraction > 70% (frequently)

^*^ Included studies provided the degree of change in a quantitative way or stated that results were within normal limits

*MGS* memory-guided saccades, *VGS* visually-guided saccades

##### Saccadic Eye Movements (SEM)

The majority of studies reported on visually-guided saccades (VGS) (14/22 studies, 98 patients), being recorded either in the horizontal plane only (n = 8 studies) or in both the horizontal and vertical plane (*n* = 6 studies). Memory-guided saccades (MGS) were obtained in three studies only (see Table 2 for details).

##### Saccade Latency in Visually-Guided Saccades

Horizontal saccade latency was found to be within normal range in all four studies (*n* = 36 patients) that assessed this parameter [17, 18, 24, 25], whereas none of the studies assessed vertical VGS latency.

##### Saccade Accuracy in Visually-Guided Saccades

Nine studies in total (*n* = 66 patients) reported on the accuracy of VGS in SCA6, describing dysmetric horizontal saccades in 36/66 patients (55%). Hypometric saccades were more frequently observed (16/48) [15–17, 19, 22, 25] than hypermetric saccades (7/53) [15–19, 22, 25]. Concerning the level of abnormality of saccade accuracy (as assessed by differences in saccadic gain), differences were usually mild and significant (compared to healthy controls) in three studies only (ranging from 0.78 ± 0.04 to 0.90 ± 0.03 for amplitudes between 2° and 30°) [15, 17, 18], whereas in the other studies differences were either not significant [19, 25] or no comparisons to healthy controls were provided [16, 22].

A single study that assessed vertical VGS accuracy found upward saccades to be significantly hypometric compared to controls (0.69 ± 0.11 vs. 0.83 ± 0.08, *p* < 0.05), whereas downward saccades were accurate (0.99 ± 0.24 vs. 0.87 ± 0.09, *p* > 0.05) [17].

##### Saccade Velocity in Visually-Guided Saccades

Peak saccade velocity for horizontal VGS was within normal limits in 39/44 SCA6 patients assessed in six studies [17–19, 22, 25, 26]. In those patients with reduced saccade velocities, changes were usually mild [17, 26]. None of the studies providing a statistical comparison of peak saccadic velocity for horizontal VGS to healthy controls found a significant decrease in the patients [18, 19, 25], whereas in two studies single subjects had significantly reduced peak saccadic velocity compared to the healthy controls [15, 17] and in another study 2/5 patients had “abnormal values” without further specifying normative values [26].

In comparison to horizontal saccades, reductions in peak velocity for vertical VGS were more frequently found (36%, 10/28 patients) and also more pronounced [15, 17, 22, 26]. For single subjects, significant reductions for downward saccadic velocity (9/28 patients) and upward saccadic velocity (9/28) were found equally frequently [15, 17, 26].

##### Memory-Guided Saccades

Horizontal MGS were assessed in a single study with a total of 12 SCA6 patients only [25]. Both MGS latency and amplitude in the SCA6 patients were within normal limits.

### Saccadic Intrusions

Saccadic intrusions (SI) were present in 23/40 patients studied (58%, from 5 studies [15, 17, 18, 26, 27]). Only square-wave jerks (SWJ) were noted, whereas no ocular flutter was described. Prevalence of SWJ amongst studies varied substantially, ranging from 100% [15, 18] to 17% [27]. Distinct patterns in SWJ presentation were noted in one study, with some subjects having a higher frequency and amplitude of SWJ, whereas others presented with higher velocity SWJ [15]. No information was provided on the amplitude of square-wave jerks in the other studies.

#### Nystagmus in Primary Gaze and Eccentric Gaze

Spontaneous nystagmus (SN) was assessed in six studies with a total of 55 patients [16, 17, 21, 22, 26, 27]. The SN pattern found was downbeat nystagmus (DBN) in 34/55 (62%) cases, horizontal unidirectional SN in 8/55 cases and horizontal periodic alternating nystagmus (PAN) in 2/55 cases. Horizontal gaze-evoked nystagmus (GEN) was also a frequent finding, being present in 48/70 (69%) of patients (data from 7 studies [16–18, 21, 22, 26, 27]), whereas rebound nystagmus (RBN) was less often reported (38% (5/13) of patients, 3 studies [17, 18, 21]). There was no information provided about the amplitude or slow-phase velocity of either SN or gaze-evoked nystagmus.

#### Triggered Nystagmus – Positional Nystagmus (PN) and Head-Shaking Nystagmus (HSN)

While positional testing was performed in only three studies, the majority of patients examined demonstrated PN (74%, 25/34). PN-patterns observed depended on the positional maneuver applied. Specifically, in supine-roll testing horizontal apogeotropic PN (*n* = 12) was more frequent than horizontal geotropic PN (*n* = 3) [16, 22], whereas in straight-head hanging position or during the Dix-Hallpike maneuver positional DBN (*n* = 8) was more often observed than positional upbeat nystagmus (UBN) (*n* = 1) [16]. In another study positional DBN (*n* = 5) or UBN (*n* = 3) was reported, but no information on positional maneuvers applied was provided [27].

Following horizontal head shaking, 55% (12/22) of SCA6 patients demonstrated horizontal HSN [22, 27] and in 62% (21/34) of patients also a “perverted” vertical HSN was observed [16, 22, 27].

### Vestibulo-Ocular Reflex and Optokinetic NYSTAGMUS (OKN) findings

#### Low-Frequency aVOR, aVOR-Suppression and Response to Caloric Irrigation

**Fig. 3:**
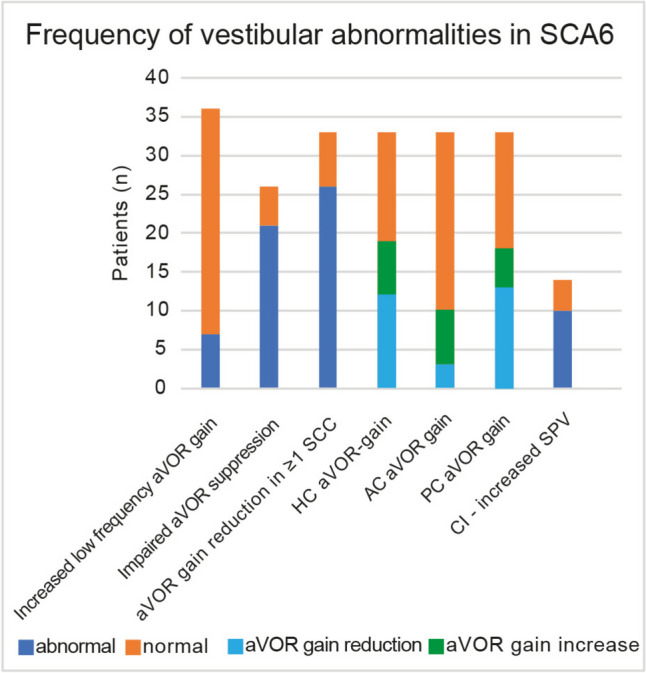
Frequency of vestibular abnormalities amongst all SCA6 patients included. Thereby the number of SCA6-patients with abnormal (in blue) and normal (in red) findings for each vestibular parameter is shown. Different properties of the angular vestibulo-ocular reflex (aVOR) were grouped (low-frequency aVOR, aVOR suppression, high-frequency aVOR and caloric irrigation). Abbreviations: AC = anterior canal; aVOR = angular vestibulo-ocular reflex; CI = caloric irrigation; HC = horizontal canal; PC = posterior canal; SCC = semicircular canal; SPV = slow-phase velocity

Low-frequency aVOR gain abnormalities (as obtained by rotatory chair testing) were observed in a minority of SCA6 patients only (7/36, 19%, 7 studies), but were seen over a broad range of frequencies (0.05 Hz-0.4 Hz) and peak velocities (30–60 deg/sec), demonstrating a pattern of abnormally high gains in all cases (see Figure 3 and Table 4). Additionally, an abnormally short time constant was measured in 6/6 patients in a single study [26]. Translational VOR gain was studied in a single study only with five patients [23], being severely reduced in all patients evaluated.

**Table 4:** Overview of vestibulo-ocular reflex findings – level of abnormality and frequency of presentation

	Level of abnormality*	*n*	Frequency of presentation	*n*
Low-frequency aVOR				
Increase in gain	↑↑ [18–20, 23, 26, 28, 43]	36	= / + [18–20, 23, 26, 28, 43]	36
Time constant	NR	NR	+ + + [26]	6
Optokinetic nystagmus				
OKN impairment—gain	↓↓↓ [18, 19, 21]	12	+ + + [18, 19, 21]	12
aVOR suppression				
Impaired gain	↓↓↓ [18–20]	14	+ + + [16, 18–20, 26]	26
High-frequency aVOR (qHIT)				
Gain reduction in at least one SCC	NR	NR	+ + + [16, 22, 28]	33
HC gain reduction	↓—↓↓ [16, 22, 28]	33	+ [16, 22, 28]	33
HC gain increase	↑ [16, 22, 28]	33	+ [16, 22, 28]	33
AC gain reduction	↓ [16, 22, 28]	33	= / + [16, 22, 28]	33
AC gain increase	↑ [16, 22, 28]	33	+ [16, 22, 28]	33
PC gain reduction	↓—↓↓ [16, 22, 28]	33	+ [16, 22, 28]	33
PC gain increase	∅ [16, 22, 28]	33	∅ [16, 22, 28]	33
Translational VOR				
Decrease in gain	↓↓↓ [23]	5	+ + + [23]	5
Caloric irrigation				
Increase in SPV	NR	NR	+ + + [21, 28]	14

Symbol legend:

Level of abnormality:

↑/ ↓ = reduced/increased by 5–25% (mildly)

↑↑/↓↓ = reduced/increased by 25–50% (moderately)

↑↑↑/↓↓↓ = reduced/increased by > 50% (strongly)

Frequency of presentation:

∅ = not present

= / +  = fraction 5–20% (rarely)

+  = fraction 20–40% (sometimes)

+  +  = fraction 40–70% (in a significant fraction)

+  +  +  = fraction > 70% (frequently)

^*^ Included studies provided the degree of change in a quantitative way or stated that results were within normal limits

Abbreviations:* AC* anterior canal, *aVOR* angular vestibulo-ocular reflex, *HC* horizontal canal, *OKN* Optokinetic nystagmus, *PC* posterior canal, *qHIT* quantitative head-impulse test, *SCC* semicircular canal, *SPV* slow-phase velocity, *vHIT* video head-impulse testing

Furthermore, impairments in aVOR-suppression (21/25 (84%) patients in 5 studies) were frequently reported over a broad range of frequencies (0.05 Hz-0.4 Hz), being usually severe. Response to caloric irrigation was hyperactive (i.e., presenting with abnormally high slow-phase velocity) in the majority in a small group of SCA6 patients studied (10/14, (84%) [21, 28], with peak eye velocities being larger than 155 deg/sec [28].

Horizontal OKN was studied in few patients and studies (*n* = 3) only and for a single stimulus frequency and peak velocity (frequency = 0.05 Hz, PV = 60 deg/sec). However, when assessed, severe OKN gain reductions were found in almost all patients (11/12, 92%) [18, 19, 21].

#### Quantitative Head-Impulse Testing (qHIT)

When assessing the high-frequency aVOR (using either search coils [28] or video-head-impulse testing (vHIT) [16, 22]) a significantly reduced aVOR-gain in one or more semicircular canal was observed in 26/33 (79%) SCA6 patients. While some patients had significantly *reduced* aVOR-gains in the horizontal canal (HC, *n* = 21/33), anterior canal (AC, *n* = 3/33) or posterior canal (PC, *n* = 13/33), other patients demonstrated significantly *increased* aVOR-gains in the horizontal (*n* = 7/33) or anterior (*n* = 7/33) canal. Noteworthy, none of the 33 patients had significantly increased PC aVOR-gains.

At the level of single studies, one study reported significantly reduced average (± 1 standard deviation (SD)) vHIT-gains only for the PCs compared to healthy controls (0.68 [0.57–0.83] vs 0.91 [0.84–0.96], *p* < 0.001) [22]. Another study found significantly larger aVOR gains for the ACs compared to those for the PCs (median = 1.07, interquartile range [IQR] = 0.98–1.34 vs. median = 0.89, IQR = 0.63–0.96, *p* = 0.005) [16], but did not provide any comparisons to healthy controls. The third study did not provide any comparisons [28].

In a meta-analysis of individual aVOR-gain values of all six canals (values from left and right side pooled for better comparison) from three studies [16, 22, 28], average (± 1SD) aVOR-gains for the horizontal (0.86 ± 0.24, range = 0.26–1.27), anterior (0.92 ± 0.18, range = 0.55–1.29) and posterior (0.74 ± 0.23, range = 0.25–1.02) canals were within normal range, reflecting the broad inter-individual range of high-frequency aVOR-gain values.

One study also provided longitudinal data (individual follow-up period: 3 months to 5 years, median = 12 months) for 12 SCA6 patients, demonstrating a significant decrease in HC aVOR gains (median = 0.97, IQR = 0.85–1.06 vs. 0.93, 0.76–0.97, *p* = 0.008) and AC aVOR gains (median = 1.07, IQR = 0.98–1.34 vs. 0.96, 0.90–1.15, *p* = 0.021) at follow-up, whereas aVOR-gains of the PCs remained unchanged (median = 0.89, IQR = 0.63–0.96 vs. 0.79, 0.48–0.88, *p* = 0.212) [16].

### Oculomotor and Vestibular Findings in Pre-Symptomatic Carriers

Quantitative oculomotor data on pre-symptomatic carriers was reported in a single study [15]. Peak velocity of upward VGS was significantly reduced in 1/4 pre-symptomatic carriers and preserved in 3/4, whereas all four carriers demonstrated downward VGS peak velocity within normal limit. This carrier also had hypometric horizontal and downward saccades, whereas saccade metrics were normal in the other three carriers. All four pre-symptomatic carriers demonstrated SWJ. Upward pursuit gain was reduced in one pre-symptomatic carrier, whereas in the other three carriers either only a trend for reduced vertical pursuit gains were noted (*n* = 2) or gains were within normal range (*n* = 1). Overall, the pattern of abnormality described in the pre-symptomatic carriers included reduced VGS velocity, reduced PEM gains and reduced gaze stability (resulting in SWJ) [15].

### Correlations Between Oculomotor / Vestibulo-Ocular Reflex Parameters and Other Abnormalities in SCA6

This systematic review also aimed to examine any associations between oculomotor / vestibulo-ocular reflex abnormalities and other relevant parameters. The range of correlations identified included age at symptom-onset, disease duration, genetic testing and clinical scale scores (ICARS, SARA).

In a single study that reported on pooled results from 12 SCA6 patients an eight SCA31 patients, significant correlations were found between various VGS and MGS parameters and ICARS, as described in detail in Table 5.

**Table 5:** Correlations: saccadic eye movements (Spearman rho values)

	Visually guided saccades (VGS)					
	% of overshoot of VGS	ratio of success trials for VGS	VGS horizontal latency (10°)	VGS horizontal latency (20°)	coefficient of variation of latency of VGS 10°	coefficient of variation of latency of VGS 20°
ICARS	0.61**, § [25]	−0.64**, § [25]	0.67**, § [25]	0.60**, § [25]	0.68**, § [25]	0.75**, § [25]
	Memory guided saccades (MGS)					
	ratio of success trials for MGS	MGS horizontal latency (10°)	MGS horizontal latency (20°)			
ICARS	−0.52*, § [25]	0.54*, § [25]	0.55*, § [25]			

Values in bold are significant

^*^P ≤ 0.05, **P ≤ 0.01

^§^ For this correlation analysis data from SCA6 (*n* = 12) and SCA31 (*n* = 8) was pooled. A comparison was made in saccade parameters between SCA6 and SCA31 and except for the coefficient of variation of amplitude in 10° target trials (being significantly larger in SCA6 than in SCA31 (*p* = 0.01)), none of the other parameters showed differences

between the two types of SCA patients

Abbreviations:* MGS* memory-guided saccades, *ICARS* International Cooperative Ataxia Rating Scale, *VGS* visually-guided saccades

All three studies reporting on qHIT gains provided correlation analyses. Two out of three studies reported significant negative correlations between qHIT aVOR gains and disease severity. While in one study a negative correlation between HC, AC and PC aVOR gains (as measured by vHIT) and the SARA score was observed (see Table 6) [16], another study identified a negative correlation between HC aVOR gains (as measured by search coils) and the ICARS (Spearman rho = –0.927, *p* < 0.001) [28]. This study also reported on low-frequency aVOR gains (as assessed by caloric irrigation and rotatory chair testing), but did not find any significant correlations with the ICARS. In a third study, no significant correlations between aVOR gains (as measured by vHIT) and the SARA score were found for both HC, AC and PC [22].

**Table 6:** Correlations: aVOR (Spearman rho values)

	High-frequency aVOR gain (qHIT)			Low-frequency aVOR gain (calorics and rotatory chair test)
	Horizontal canal	Anterior canal	Posterior canal	Horizontal canal
Age	ns [16, 28]	ns [16, 28]	ns [16, 28]	ns [28]
Age at symptom onset	ns [22, 28]	ns [22, 28]	ns [22, 28]	ns [28]
CAG repeat lenght	ns [16, 22, 28]	ns [16, 22, 28]	ns [16, 22, 28]	ns [28]
Disease duration	ns [16]	ns [16]	ns [16]	ns [28]
ICARS	−0.93** [28]	ns [28]	ns [28]	ns [28]
SARA	−0.68** [16], ns [22]	−0.64** [16], ns [22]	−0.61** [16], ns [22]	-

Values in bold are significant

^*^ *p* ≤ 0.05, ** *p* ≤ 0.01

Abbreviations:* aVOR* angular vestibulo-ocular reflex, *ns* non-significant, *ICARS* International Cooperative Ataxia Rating Scale, *SARA* Scale for the Assessment and Rating of Ataxia, *qHIT* quantitative head-impulse test

No significant correlations were reported between HC, AC and PC high-frequency aVOR gains and CAG-repeat expansion size [16, 22, 28], age at onset [22, 28], disease duration [16], and age [16, 28]. Likewise, no significant correlations were found for low-frequency aVOR-gains (as measured by caloric irrigation and rotatory chair testing) and CAG-repeat expansion, age, age at disease onset and disease duration in one study [28].

## Discussion

The main goal of this systematic review was to provide a comprehensive overview of oculomotor and vestibular deficits observed in SCA6 patients by use of quantitative measurements, as well as to highlight those abnormalities most suitable for facilitating early detection and diagnosis and for monitoring disease progression in natural history studies and symptom improvement in treatment trials. The most frequently identified and most severe eye movement abnormalities in SCA6 patients were deficits in PEM gain (84%), impaired aVOR-suppression (84%) and high-frequency aVOR-deficits (79%), whereas SEM were only mildly affected and often within normal limits. We identified no data on the value of quantitative oculomotor/vestibular measurements in treatment trials and information on the value of quantitative oculomotor/vestibular assessments in pre-symptomatic carriers and for longitudinal studies was scarce. Overall, the range of oculomotor and vestibular abnormalities observed in SCA6 patients likely reflects mostly central vestibular disease. Specifically, the presence of DBN and GEN, perverted HSN, gain reduction in PEM, aVOR-suppression impairment, and SI point to central (cerebellar) involvement. Likewise, the hyperactive responses to caloric irrigation and the altered (either increased or reduced) vHIT-gains may indicate cerebellar floccular dysfunction rather than peripheral deficits [16, 22, 28]. Oculomotor impairments as seen in CA have been linked to damage to the oculomotor cerebellum in lobules IX and X (flocculus, paraflocculus, nodulus in the vermis) and the vermal lobule VII [29].

### Proposed Oculomotor / Vestibular Paradigms in SCA6 Patients

Based on the systematic review and meta-analysis performed, a fingerprint of oculomotor and vestibular deficits in SCA6 can be defined (see Table 7 for details). While a broad range of paradigms has shown abnormalities in SCA6 patients, the frequency and the magnitude of the abnormalities varied (see Tables 3 and 4). Thus, focusing on the paradigms that have been associated with frequent and pronounced alteration in SCA6 patients is recommended. Such a “core” set of oculomotor and vestibular paradigms should include an assessment of saccadic eye movements (focusing on VGS metrics), pursuit eye movements (focusing on gain), gaze holding at primary gaze (looking for DBN) and eccentric gaze (evaluating for GEN), triggered nystagmus (focusing on positional testing and HSN) and aVOR-responses (gain reduction) assessed by VOR suppression and vHIT. Specific parameters for measuring these domains are straight-forward and in accordance with those previously published by our group [6, 8]. Gaze instabilities are best caught during gaze straight-ahead (for at least 60 s) and at horizontally eccentric gaze (5–30° eccentricity, kept for at least 10 s) using a flashing target, pursuit eye movements should be assessed using sinusoidal stimuli with 0.1–0.4 Hz frequency and 10–20 deg amplitude (center to peak) in the horizontal and vertical plane, visually-guided saccades should be measured using stimulus amplitudes in the range of 10–30 deg in the horizontal and vertical plane. Positional testing is preferentially performed by both using the supine-roll test, straight head-hanging and the Dix-Hallpike maneuver. For the assessment of HSN horizontal head shaking should be applied (proposed paradigm: frequency = 2–3 Hz, amplitude =  ± 10 deg, duration = 15 s [30]). For VOR-suppression sinusoidal stimuli with frequencies of 0.05–0.2 Hz and peak velocities of 12–60 deg/sec should be used. For video head-impulse testing normative values have been provided previously [31, 32].

**Table 7:** SCA6 oculomotor / vestibular “fingerprint”

Domain	Key oculomotor / vestibular changes
Pursuit eye movements	**• Horizontal and vertical pursuit gain severely reduced** [15–22]
Saccadic eye movements	
Memory-guided saccades	**•** Preserved horizontal latency [25] **•** Preserved horizontal accuracy [25]
Visually-guided saccades	**•** Saccadic latency within normal limits [17, 18, 24, 25] **• Frequent, but mild horizontal saccadic dysmetria** [15–19, 21, 22, 25, 27] **•** Rarely slightly reduced horizontal PV, sometimes moderately reduced vertical PV [17–19, 22, 25, 26]
Saccadic intrusions	**• Frequent SWJ** [15, 17, 18, 26, 27]
Spontaneous nystagmus	**• Frequent DBN** [16, 17, 21, 22, 26, 27] **•** Rarely horizontal nystagmus [16, 17, 21, 22, 26, 27]
Gaze-holding (eccentric gaze)	**• GEN** in a significant fraction [16–18, 21, 22, 26, 27] **•** Sometimes rebound nystagmus [17, 18, 21]
Triggered nystagmus	**• Frequently horizontal HSN and vertical (“perverted”) HSN** [16, 22, 27] **• Very frequently positional nystagmus** [16, 22, 27] **• Very frequent and severe OKN gain reduction** [18, 19, 21]
Angular vestibulo-ocular reflex (VOR)	
Caloric irrigation	**• Increased aVOR gains very frequently observed** [21, 28]
High-frequency aVOR (qHIT)	**• Impairment of at least one SCC very frequently observed** [16, 22, 28] **• Preferential impairment of PC aVOR gains** [16, 22]
Low-frequency aVOR (rotatory chair testing)	**•** Usually normal, rarely increased aVOR gain [18–20, 23, 26, 28, 43]
aVOR suppression	**• Strongly impaired in almost all patients** [16, 18–20, 26]

Abbreviations: *AC* anterior canal, *aVOR* angular vestibulo-ocular reflex, *DBN* downbeat nystagmus, *GEN* gaze-evoked nystagmus, *HC* horizontal canal, *HSN* head-shaking nystagmus, *MGS* memory-guided saccades, *OKN* optokinetic nystagmus, *PC* posterior canal, *PV* peak velocity, *qHIT* quantitative head-impulse test, *SI* saccadic intrusions, *SWJ* square-wave jerks, *VGS* visually-guided saccades, *vHIT* video-head-impulse test

### Oculomotor / Vestibular Fingerprint in SCA6 – Overlap with Other Hereditary Ataxias

Considering the oculomotor and vestibular profile identified in SCA6, various other hereditary ataxias may present similarly. Thus, differentiating SCA6 from other cerebellar ataxias is essential. While pursuit gain reductions are typically severe in SCA6, gain reductions may also be observed in ataxia telangiectasia (A-T), but the distinct age at symptom-onset and other clinical features usually allow for a reliable distinction. Likewise, reduced pursuit gain may also be observed in Niemann-Pick disease type C (NPC) [6, 8]. In general, abnormalities in SEM were infrequent and usually mild in SCA6, resulting in minor dysmetria or slightly reduced VGS velocities only. Thus, important differential diagnoses are SCA3 (with saccade latency being preserved as well) [6], whereas in FRDA [10], A-T and SCA2 moderately increased saccadic latencies were observed. Severely slowed saccades are the hallmark sign of SCA2 and for NPC when affecting primarily the vertical plane [6, 8], but mild SEM abnormalities are frequently seen and thus of limited value in the differential diagnosis of SCA6. SWJ are also frequently identified in Friedreich ataxia (FRDA, 89%) [10], SCA3 (43–64%), A-T (31–85%) [6], and in SCAR4 (formerly SCASI and SCA24) [33]. In contrast, SCA1 and SCA2 patients less often display SWJ [6] (20–30%). With DBN being frequently found in SCA6, important differential diagnoses include SCA17, SCA27B [34], NPC, and CANVAS/RFC-1 related disorders [6, 35], whereas it is less often observed in SCA1, SCA2 and SCA3 [22] and FRDA [10].

Evaluating for the presence of triggered nystagmus such as positional nystagmus or head-shaking nystagmus seems promising in SCA6 and may allow for the differentiation from other SCAs. Specifically, in a single study PN and (perverted) HSN was identified only in SCA6 patients, whereas SCA2, SCA3 and SCA7 patients had normal findings [22]. However, for many other cerebellar ataxias there is no quantitative data available for these parameters.

Focusing on high-frequency aVOR gains, which can be readily and reliably quantified using vHIT, studies have reported a characteristic pattern of aVOR-gain changes in SCA6. Specifically, predominant reductions in posterior-canal (PC) aVOR-gains were found in two studies, with the horizontal canal (HC) and anterior canal (AC) function relatively spared [16, 22]. Importantly, those SCA6 patients more mildly affected (based on ICARS) often presented with gain increases, whereas those patients more severely affected demonstrated aVOR-gain reductions in one study [28]. In contrast, vHIT-gain reductions were more widespread in SCA3, affecting both HC, AC and PC, or involved mainly the AC as seen in SCA7 [22]. For SCA27B (GAA-FGF14-related ataxia), recently moderately reduced horizontal aVOR-gain values were described [34, 36], whereas no information is available on vertical canal function in SCA27B. In comparison to the severe aVOR-gain reductions seen in RFC1-related ataxia (CANVAS syndrome) [36], impairments observed in SCA6 are much milder, thus differentiation is usually straight-forward. In FRDA, moderate HC vHIT-gain reductions were reported in two studies [37, 38], whereas no information on vertical canals was available. In SCA1 aVOR-gains are typically preserved [38], whereas in SCA2 aVOR-gain reductions were either mild only [38] or aVOR gains were within normal range [22]. Thus, the pattern of vHIT-gain alterations may be valuable in the differential diagnosis of CA, but disease stage may significantly affect aVOR gains. While aVOR-suppression and OKN-responses were markedly impaired in SCA6, these findings are non-specific and thus of limited value for the differential diagnosis.

### Early Disease Detection, Measurement of Disease Progression and Disease Severity

Few studies reported on correlations between disease severity and selected oculomotor/vestibular parameters. Whereas two different scales for assessing disease severity were considered (ICARS, SARA), comparisons were restricted to aVOR gains (high-frequency and low-frequency) [16, 22, 28] and saccadic eye movements (VGS, MGS) [25]. With a significant moderate-to-strong correlation between horizontal aVOR gains as assessed by quantitative head-impulse testing and SARA [16] and ICARS [28], respectively, this parameter may serve as a surrogate biomarker for assessing disease severity in SCA6. Furthermore, significant correlations were reported between both VGS latency and accuracy and disease severity (ICARS) and between MGS latency and disease severity in a single study that pooled results from SCA6 and SCA31 patients, albeit stating that for all but one parameter no significant differences between SCA6 and SCA31 patients were found [25]. With overall changes in SEM in SCA6 being mild and with latency overall remaining within normal limits, the impact of these correlations remains unclear and needs further evaluation. There were no correlations between clinical parameters (age, age at symptom onset, disease duration) and aVOR gains, which may be explained by the substantial variability in age at symptom onset, disease severity, and disease progression in SCA6 [39] and likely also by the small sample size (*n* = 12–23 patients) in our systematic review for this parameter. Likewise, CAG repeat expansion size did not correlate with aVOR-gains in three studies including a total of 33 patients [16, 22, 28], being in agreement with recent data indicating no correlation between CAG repeat expansion size and the SARA score in a SCA6 cohort with 125 patients [40].

Likewise, information on suitable quantitative oculomotor / vestibular parameters for assessing disease progression in SCA6 is scare. We identified a single study only that obtained follow-up assessments of quantitative oculomotor function, reporting significant decreases in aVOR gains (vHIT) for both the HC, AC and PC over a period of three months to five years [16]. Overall, obtaining serial vHIT measurements seems most suitable to both assess disease severity and monitor disease progression in SCA6 patients. vHIT measurements are also now readily available, are well-defined with regards to the paradigm and its interpretation and can be reliably and relatively easily applied by trained technicians.

Information on the clinical presentation in pre-symptomatic mutation carriers as assessed by quantitative oculomotor paradigms was found only in a single study which included four patients in our review [15], thus little is known about early digital oculomotor markers in SCA6. Current evidence suggests that presence of SWJ, upward pursuit gain reductions, decreased horizontal and downward saccade accuracy, and decreased upward saccade velocity may be early, subtle oculomotor signs in pre-symptomatic carriers. This, however, should be validated in a larger patient cohort.

### Limitations

Comparison of oculomotor and vestibular test results amongst studies is limited by differences in the measurement methods. This was present at several levels: different eye-movement recording equipment, varying combination of plains measured (horizontal or horizontal and vertical) and discordance in the specific conditions such as stimulus velocity or saccade amplitudes. This was reflected also in a moderate or low overall study quality in the majority of the studies (15/22). Given this heterogeneity, a meta-analysis across studies was possible only for carefully selected paradigms such as high-frequency aVOR gains. Furthermore, details on recording parameters and definitions of eye movement types studied were sometimes vague or even lacking (as e.g. for SWJ or for VGS recording [basic step paradigm vs. gap paradigm]) and possibly resulting in wrongly classified eye movement recordings. The almost complete absence of longitudinal studies and data on pre-symptomatic carriers (one single study identified only for each condition) confines our knowledge about suitable oculomotor parameters to monitor early disease and disease progression. These factors underscore the need for standardized data collection methods and longitudinal observational studies to better understand SCA6 and its associated eye movement abnormalities.

## Conclusions

In our systematic review and meta-analysis on quantitative oculomotor / vestibular parameters in SCA6 we identified a characteristic – albeit not pathognomonic – disease pattern. The most frequently observed eye movement abnormalities in SCA6 patients were deficits in PEM gain (84%), impaired aVOR-suppression (84%), high-frequency aVOR-deficits (79%), positional nystagmus (74%), gaze-evoked nystagmus (69%), DBN (62%) and perverted (vertical) nystagmus after horizontal head-shaking (62%). For horizontal visually-guided saccades (VGS), changes in metrics (55%) were frequently observed but usually mild, whereas saccade velocity was usually preserved and saccade latency within normal limits. Thus, substantial overlap to other hereditary ataxias including FRDA, SCA3, SCA27B, and CANVAS exists. With perverted (vertical) HSN being not observed in other SCAs (SCA2, 3, and 7) in one study, this oculomotor parameter seems a promising oculomotor biomarker for the differential diagnosis, but this awaits confirmation in future studies. Obtaining serial vHIT measurements seems most suitable to both assess disease severity and monitor disease progression in SCA6 patients at this time (as summarized in Table 8), however, data on oculomotor / vestibular impairment in early (pre-symptomatic) stages and longitudinally is scare. Including the Scale for Ocular motor Disorders in Ataxia (SODA), the impact of eye movement abnormalities in cerebellar ataxias could be monitored as well in SCA6 [41]. The need for the standardization of data collection methods and longitudinal observational studies is paramount. Only then we will be able to better understand and accurately study SCA6 and its associated eye movement abnormalities.

**Table 8:** The value of oculomotor / vestibulo-ocular reflex parameters as biomarkers in SCA6

Clinical assessment	Parameters
Potential markers for quantifying disease severity	• High-frequency aVOR gain (vHIT), being increased in mildly affected cases and reduced (especially PC) for more severely affected cases [16, 22, 28] • Horizontal and vertical PEM demonstrating progressively reduced pursuit gain [15–22] • Horizontal and vertical SEM demonstrating progressive dysmetria [15–19, 21, 22, 25, 27] and/or slowing [17–19, 22, 25, 26]
Potential markers for monitoring disease progression	• Repeated high-frequency aVOR gain measurements (vHIT), demonstrating progressive individual gain reductions over time [16]

Abbreviations:* AS* anti-saccades, *aVOR* angular vestibulo-ocular reflex, *DBN* downbeat nystagmus, *FRDA* Friedreich Ataxia, *MGS* memory-guided saccades, *PEM* pursuit eye movements, *SEM* saccadic eye movements, *SWJ* square-wave jerks, *VGS* visually-guided saccades, *vHIT* video-head-impulse test

## Supplementary Information

Below is the link to the electronic supplementary material.

Supplementary file1 (DOCX 119 KB)

## Data Availability

The data that support the findings of this study are available from the corresponding author upon reasonable request.
