# Supplementary material for: Quantitative Oculomotor and Vestibular Profile in Spinocerebellar Ataxia Type 6 – Systematic Review and Meta-Analysis
Source: Cerebellum. 2024 Dec 15;24(1):12. doi: 10.1007/s12311-024-01774-y (PMC11646955; doi:10.1007/s12311-024-01774-y)
Supplement: Supplementary file 1 — Supplementary file1 (DOCX 119 KB) [file 12311_2024_1774_MOESM1_ESM.docx]

## Online-Only Supplemental Materials

## Appendix 1: Electronic search strategy, coding-scheme for the systematic review and data analysis

**The search strategy was designed by a clinical investigator with relevant domain expertise in neurology (AAT). This search string has been applied in a previous publication from this group [1] and has been re-run for this follow-up study on May 7^th^ 2024, thus covering also the period after May 13^th^ 2021.**

**We searched MEDLINE for English-language articles, using the following strategies with the following components: (1) defining the clinical syndrome (i.e., ataxia), (2)** ocular motor or vestibular features**, and (3) quantitative assessments. We did not expressly search for hereditary ataxia syndromes,** as this was found to result in omitting most relevant studies because they did not refer to the genetic background in a uniform fashion**. We also performed a manual search of reference lists from eligible articles, and contacted corresponding authors where necessary. We did not seek to identify research abstracts from meeting proceedings or unpublished studies.**

MEDLINE Search *(accessed via PubMed at www.ncbi.nlm.nih.gov/pubmed)*

(ataxia OR ataxic OR (gait AND impairment)) AND ((eye movements) OR (ocular motor) OR oculomotor OR vestibular OR saccade OR (smooth pursuit) OR (vestibulo-ocular reflex) OR VOR OR optokinetic OR nystagmus OR gaze OR head impulse OR caloric) AND (quantitative OR recording OR recordings OR quantified OR measured)

Search Results

Our search identified 988 unique citations, of which 755 (76.4%) were excluded at the abstract level (Figure 1, main manuscript). A record was excluded only if two scorers (PG, AAT) recommended exclusion (detailed list of predefined reasons for exclusion shown below). We did not demand concordance on reason for abstract exclusion, but, among those abstracts with concordant reasons for exclusion (62.8%, n=474), the distribution was as follows: 41.9% had no data on human subjects with ataxia**; 7.3% were not reporting on the assessment of oculomotor /vestibular features; 6.6% were not reporting on quantitative measurements; 6.5% had no original data and 0.5% were not in English.**

We sought to examine 233 full manuscripts (this included 17 articles identified by hand-search). After initial screening, there were a total of 17 disagreements about study inclusion for the two reviewers (PG and AAT, kappa=0.84 [95% CI: 0.80 – 0.89]). These differences were resolved by discussion. Overall agreement on reason for exclusion was 94%. In order to achieve concordance on reason for full-text exclusion, we resolved differences by discussion.

At the end of our full-text review, 841 were excluded and 147 were considered eligible (Figure 1, main manuscript). These eligible studies represented 14.9% of the total (n=988). Among all full-text manuscripts excluded (37.2%), the distribution of reason for exclusion was as follows: 22.6**% were not reporting on quantitative measurements;** 9.0% had no data on human subjects with ataxia**; 2.6% were not reporting on the assessment of oculomotor /vestibular features; 2.6% were not in English and 0.4% had no original data.**

**While the previous steps of the literature search were identical to those of an ongoing study on suitable digital oculomotor markers in various hereditary ataxias (in preparation, Pretegiani et al. 2024), we focused on publications reporting on genetically-confirmed SCA6 in this review. Thus, from the 147 manuscripts identified after the full-text review, only the 22 publications (2.2%) reporting on patients with** genetically-confirmed spinocerebellar ataxia type 6 (SCA6) were selected.

The details of the coding schema for abstract and full-text reviews have been previously been described in detail [1].

## Appendix 2: quality assessment for studies reporting on oculomotor findings in ataxia

Two independent reviewers (PG and AAT) rated all included studies with regards to their quality in reporting oculomotor testing in ataxia patients. Discrepancies were resolved by discussion. Based on the eight items listed below an overall quality rating (high, moderate, low) was assigned. “High quality” was defined as having high quality on items 1, 3-7 and a low risk-of-bias for item 8. Item 2 was not considered since, depending on the study design a control group may not be required (e.g. for treatment response studies). “Moderate quality” studies were defined as studies having at least moderate quality ratings for all three items (with „low“ or „high“ risk of bias for item 8). All studies that received a low quality rating in one or several items (items 1-7) or had an unknown risk of bias for item 8 were considered “low quality” studies. This rating has been previously described, the detailed criteria for the quality assessment can be found in these previous publications [1, 2].

# Appendix 3 – additional tables

Supplementary Table 1 – quality assessment for studies reporting on oculomotor findings in ataxia – overview on all selected studies

| **Table S1: quality assessment for studies reporting on oculomotor findings in ataxia – overview on all selected studies reporting on SCA6 patients** | | | | | | | | | |
| --- | --- | --- | --- | --- | --- | --- | --- | --- | --- |
| **Study** | **Patient selection** | **Control group selection** | **Rigidity of recording protocol applied** | **Description of recording parameters used** | **Appropriateness of recording devices used for OM /vestibular testing** | **Appropriateness of data analysis applied** | **Assessment of obtained test results for significance** | **Risk of bias for assessing test results** | **OVERALL study quality rating** |
| Bour et al. 2008 [3] | high | moderate | high | high | high | high | high | low | high |
| Büttner et al. 1998 [4] | high | not applicable | high | high | moderate | high | low | high | moderate |
| Chang et al. 2020 [5] | low | low | high | high | moderate | high | high | low | low |
| Christova et al. 2008 [6] | high | moderate | high | high | high | moderate | high | low | moderate |
| Crane et al. 2000 [7] | low | low | high | high | high | high | high | low | low |
| Dakin et al. 2018 [8] | high | moderate | high | high |  | high | high | low | high |
| Gomez et al. 1997 [9] | high | not applicable | high | high | high | moderate | low | high | low |
| Hashimoto et al. 2003 [10] | high | not applicable | high | moderate | high | high | high | low | moderate |
| Huh et al. 2015 [11] | high | moderate | high | high | high | high | high | low | high |
| Inomata-Terada et al. 2023 [12] | high | moderate | high | high | high | high | high | low | high |
| Joiner et al. 2005 [13] | high | low | high | high | low | moderate | low | low | low |
| Kerber et al. 2005 [14] | high | not applicable | high | moderate | moderate | moderate | moderate | high | moderate |
| Kim et al. 2013 [15] | high | moderate | high | high | high | high | high | high | moderate |
| Kim et al. 2023 [16] | high | moderate | high | moderate | moderate | moderate | high | low | moderate |
| Lasker et al. 2005 [17] | moderate | moderate | high | high | high | moderate | high | low | moderate |
| Lee et al. 2020 [18] | high | not applicable | high | moderate | high | high | high | low | moderate |
| Matsuda et al. 2014 [19] | moderate | moderate | high | high | high | high | high | low | high |
| Matsuda et al. 2015 [20] | high | moderate | high | moderate | high | high | high | low | moderate |
| Takeichi et al. 2000 [21] | high | moderate | high | high |  | high | high | low | high |
| Terao et al. 2016 [22] | moderate | moderate | high | high | moderate | high | high | low | moderate |
| Terao et al. 2017 [23] | moderate | moderate | high | high | moderate | high | high | low | moderate |
| Wiest et al. 2001 [24] | high | moderate | high | high | high | high | high | low | high |

Supplementary Table 2 – Meta-data of included studies listed in alphabetical order

| **Table S2: meta-data of selected studies** | | | | | | | | |
| --- | --- | --- | --- | --- | --- | --- | --- | --- |
| **Author, year (citation)** | **Study population** | **Study location** | **Data collection (analysis)** | **Subjects SCA6 (% females)** | **Mean age SCA6 (SD)** | **Paradigms recorded** | **Recording device used** | **Special comments** |
| Bour et al. 2008 [3] | SCA6 and other ADCA | monocentric | Prospective, case-control-study | 5 (40%) | 62 (8) | PEM, VGS, SI, SN, HN, GEN, | Search coils |  |
| Büttner et al. 1998 [4] | SCA1, SCA2, SCA3, SCA6 | monocentric | Prospective, case-series | 5 (NR) | 59 (5) | PEM, VGS, SI, OKN, rVOR, VVOR, VORs, SN, GEN, RBN, | EOG |  |
| Chang et al. 2020 [5] | FRDA, SCA1, SCA2, SCA3, SCA6, other ADCA (not specified), AT, HSP, ARCA, MSA-C | monocentric | Prospective, case-control-study | 6 (NR) | NR | PEM | VOG (smartphone) | No information about genetic testing was provided. No findings for specific hereditary ataxia syndromes as SCA6 reported |
| Christova et al. 2008 [6] | SCA6 | monocentric | Prospective, case-control-study | Symptomatic patients=5 (60%)  Pre-symptomatic carriers=4 (50%) | Symptomatic patients=51 (7)  Pre-symptomatic carriers=32 (8) | PEM, VGS, SI, | Search coils | This study included both pre-symptomatic carriers (n=4) and symptomatic SCA6 patients (n=5) |
| Crane et al. 2000 [7] | FRDA, SCA3, SCA6, ARCA, EOCA | monocentric | Prospective, case-control-study | 11 (NR) | 48 (16) | rVOR, tVOR | Search coils |  |
| Dakin et al. 2018 [8] | SCA6 | monocentric | Prospective, case-control-study | 15 (47%) | 67 (10) | SVV | NA |  |
| Gomez et al. 1997 [9] | SCA6 | Monocentric | Prospective, case series | 13 (NR) | NR | PEM, VGS, SI, rVOR, rVOR decay Tc, VORs, SN, GEN, | Search coils |  |
| Hashimoto et al. 2003 [10] | SCA6 | monocentric | Prospective, case series | 1 (33%) | 56 (5) | PEM, VGS, OKN, SN, GEN, RBN, CI | EOG |  |
| Huh et al. 2015 [11] | SCA6 | monocentric | Prospective, case-control-study | 11 (45%) | 59 (12) | PEM, rVOR, qHIT, CI | EOG or search coils |  |
| Inomata-Terada et al. 2023 [12] | SCA 6, SCA31 | monocentric | Prospective, case-control-study | 12 (55%) | 64 (9) | VGS, MGS, | VOG (eyelink II, SR Research | Only pooled analysis (SCA6 and SCA31) presented. A comparison was made in saccade parameters between SCA6 and SCA31 and except for the coefficient of variation of amplitude in 10° target trials (being significantly larger in SCA6 than in SCA31 (p = 0.01)), none of the other parameters showed differences  between the two types of SCA patients. |
| Joiner et al. 2005 [13] | SCA6 | Monocentric | Prospective, case series | 3 (NR) | NR | VGS | VOG (NR) |  |
| Kerber et al. 2005 [14] | SCA1, SCA6, SCA8, LOCA | Monocentric | Prospective, case series | 20 (NR) | NR | PEM, VGS, OKN, rVOR, VORs | EOG |  |
| Kim et al. 2013 [15] | FRDA, SCA1, SCA2, SCA3, SCA6, SCA7, SCA8, other ADCA | monocentric | Prospective, case-control-study | 12 (33%) | 57 (13) | PEM, VGS, SI, SN, HSN, PN, GEN, | VOR (Micromedical Technologies) |  |
| Kim et al. 2023 [16] | SCA2, SCA3, SCA6, SCA7 | Monocentric | Prospective, case-control-study | 10 (50%) | 56 (11) | PEM, VGS, SI, SN, HSN, PN, GEN, qHIT | VOG (SLMed, South Korea; ICS impulse goggles, Natus for vHIT) |  |
| Lasker et al. 2005 [17] | SCA6, CA | monocentric | Prospective, case-control-study | 6 (NR) | NR | VGS | Search coils |  |
| Lee et al. 2020 [18] | SCA6 | monocentric | Retrospective, case series | 6 (50%) | 56 (13) | PEM, VGS, VORs, SN, HSN, PN, GEN, qHIT, SVV | VOG (SLMed, South Korea (also for vHIT) or SMI, Germany) | Only study with longitudinal data (vHIT), demonstrating decreased in aVOR gains for HC and AC during follow-up. aVOR gains of PC were unchanged. |
| Matsuda et al. 2014 [19] | SCA6, SCA31 | monocentric | Prospective, case-control-study | 11 (NR) | NR | SEM (visual search task) | VOG (Eyelink II, SR research) | Only a pooled analysis including both SCA6 (n=11) and SCA31 (n=7) cases is provided. |
| Matsuda et al. 2015 [20] | SCA6, SCA31 | monocentric | Prospective, case-control-study | 1 (NR) | NR | SEM (visual search task) | VOG (Eyelink II, SR research) | Only a pooled analysis including both SCA6 (n=12) and SCA31 (n=7) cases is provided.  Overlap with Matsuda et al. 2014 [19], only 1 new patient |
| Takeichi et al. 2000 [21] | SCA6 | Monocentric | Prospective, case-control-study | 5 (20%) | 57 (8) | PEM, rVOR, VVOR, VORs, | VOG (unclear) |  |
| Terao et al. 2016 [22] | SCA6, SCA8, SCA31, MSA-C, CA | Monocentric | Prospective, case-control-study | 3 (NR) | NR | VGS, MGS | EOG | Only a pooled analysis including other SCAs and 5 genetically not confirmed CA cases presented. |
| Terao et al. 2017 [23] | SCA6, SCA8, SCA31, MSA-C, CA | Monocentric | Prospective, case-control-study | 1 (NR) | NR | VGS, MGS | EOG | Overlap with Terao et al. 2016 [22], only 1 new patient  Only a pooled analysis including other SCAs and 3 genetically not confirmed CA cases presented. |
| Wiest et al. 2001 [24] | SCA6, EA2 | Monocentric | Prospective, case-control-study | 5 (NR) | 63 (11) | PEM, aVOR, tVOR | Search coils |  |

Abbreviations: ADCA=autosomal-dominant cerebellar ataxia; ARCA=autosomal-recessive cerebellar ataxia; AT=ataxia telangiectasia; CA=cerebellar ataxia (not further specified); CI=caloric irrigation; EA=episodic ataxia; EOCA=early-onset cerebellar ataxia; EOG=electro-oculography; FRDA=Friedreich Ataxia; GEN=gaze-evoked nystagmus; HN=hyperventilation nystagmus; HSN=head-shaking nystagmus; LOCA=late-onset cerebellar ataxia; MSA-C=multisystem atrophy, cerebellar type; MGS=memory-guided saccades; OKN=optokinetic nystagmus; EM=pursuit eye movements; PN=positional nystagmus; qHIT=quantitative head-impulse test; RBN=rebound nystagmus; rVOR=rotational vestibulo-ocular reflex; SCA=spinocerebellar ataxia; SEM=saccadic eye movements; SI=saccadic intrusions; SN=spontaneous nystagmus; SVV=subjective visual vertical; tVOR=translational vestibulo-ocular reflex; VOG=video-oculography; VORs=vestibulo-ocular reflex suppression; VGS=visually-guided saccades; VVOR=visually-enhanced vestibulo-ocular reflex.

## Table S3: Recording set up and normative values across studies

| Table S3: Recording set up and normative values across studies | | |
| --- | --- | --- |
|  | **Studies (n)** | **Subjects (n)** |
| **Plane of eye movement recordings** |  |  |
| horizontal plane only | 11 | 43 |
| both horizontal and vertical plane | 10 | 96 |
| no eye movement data collected | 1 | 15 |
|  |  |  |
| **Number of eyes recorded** |  |  |
| One eye | 9 | 64 |
| Both eyes | 7 | 49 |
| Unclear | 5 | 26 |
|  |  |  |
| **Technique used for eye movement recordings** |  |  |
| Scleral search coils | 6 | 40 |
| Electro-oculography (EOG) | 5 | 16 |
| Video-oculography (incl. infrared) (VOG) | 9 | 72 |
| Smartphone (240 Hz) | 1 | 6 |
| Unclear | 2 | 8 |
| Micromedical Technologies (100 Hz) | 1 | 12 |
| EyeLink II (SR Research) (1000 Hz) | 3 | 24 |
| SLMed (60Hz) for oculomotor testing and ICS Impulse (250 Hz) for aVOR (vHIT) | 1 | 10 |
| SLMed (60Hz) or SMI (recording frequency N/A) for oculomotor testing and SLMed (60Hz) for vHIT | 1 | 12 |
| Mixed (EOG or search coils) | 1 | 11 |
|  |  |  |
| **Source of normative values used*** |  |  |
| From own laboratory | 19 | N/A |
| From previous publications | 1 | N/A |
| Unclear | 2 | N/A |

Abbreviations: aVOR=angular vestibulo-ocular reflex; EOG=electro-oculography; vHIT=video head-impulse-test; VOG=video-oculography;

* Numbers of healthy control subjects included were inconsistently reported only, thus a total number of subjects is not available (N/A)

Table S4: characteristics of quantitative recordings

| **Table S4: characteristics of quantitative recordings (n=22 studies)** | | |
| --- | --- | --- |
|  |  |  |
| **Parameter** | **Studies (n)** | **Subjects (n)** |
| *Pursuit eye movements* | 11 | 76 |
| *Saccadic eye movements* |  |  |
| visually-guided SEM | 14 | 98 |
| memory-guided SEM | 3 | 16 |
| *Saccadic intrusions* | 5 | 44 |
| *Gaze holding* |  |  |
| spontaneous nystagmus | 7 | 60 |
| gaze-evoked nystagmus | 7 | 60 |
| rebound nystagmus | 2 | 8 |
| *Triggered nystagmus* |  |  |
| head-shaking nystagmus | 3 | 34 |
| hyperventilation-induced nystagmus | 1 | 5 |
| positional nystagmus | 3 | 34 |
| *OKN* | 3 | 12 |
| *VOR* |  |  |
| VOR rotational | 7 | 44 |
| VOR decay Tc | 1 | 13 |
| VVOR rotational | 2 | 10 |
| VOR translational | 2 | 7 |
| VOR suppression | 5 | 39 |
| qHIT | 3 | 33 |
| Caloric irrigation | 2 | 14 |
| *Behavioral tasks* |  |  |
| subjective visual vertical (SVV) | 1 | 15 |

Abbreviations: OKN=optokinetic nystagmus; qHIT=quantitative head-impulse test; SEM=saccadic eye movements; Tc=time constant; VOR=vestibulo-ocular reflex; VVOR=visually-enhanced VOR; VORS=vestibulo-ocular reflex suppression.

## References

[1] Garces P, Antoniades CA, Sobanska A, Kovacs N, Ying SH, Gupta AS, Perlman S, Szmulewicz DJ, Pane C, Nemeth AH, Jardim LB, Coarelli G, Dankova M, Traschutz A and Tarnutzer AA. Quantitative Oculomotor Assessment in Hereditary Ataxia: Systematic Review and Consensus by the Ataxia Global Initiative Working Group on Digital-motor Biomarkers. Cerebellum (London, England) 2023. doi 10.1007/s12311-023-01559-9

[2] Garces P, Antoniades CA, Sobanska A, Kovacs N, Ying SH, Gupta AS, Perlman S, Szmulewicz DJ, Pane C, Nemeth AH, Jardim LB, Coarelli G, Dankova M, Traschutz A and Tarnutzer AA. Quantitative Oculomotor Assessment in Hereditary Ataxia: Discriminatory Power, Correlation with Severity Measures, and Recommended Parameters for Specific Genotypes. Cerebellum (London, England) 2023. doi 10.1007/s12311-023-01514-8

[3] Bour LJ, van Rootselaar AF, Koelman JH and Tijssen MA. Oculomotor abnormalities in myoclonic tremor: a comparison with spinocerebellar ataxia type 6. Brain : a journal of neurology 2008: 131:2295-303. doi 10.1093/brain/awn177

[4] Buttner N, Geschwind D, Jen JC, Perlman S, Pulst SM and Baloh RW. Oculomotor phenotypes in autosomal dominant ataxias. Archives of neurology 1998: 55:1353-7. doi 10.1001/archneur.55.10.1353

[5] Chang Z, Chen Z, Stephen CD, Schmahmann JD, Wu HT, Sapiro G and Gupta AS. Accurate detection of cerebellar smooth pursuit eye movement abnormalities via mobile phone video and machine learning. Scientific reports 2020: 10:18641. doi 10.1038/s41598-020-75661-x

[6] Christova P, Anderson JH and Gomez CM. Impaired eye movements in presymptomatic spinocerebellar ataxia type 6. Archives of neurology 2008: 65:530-6. doi 10.1001/archneur.65.4.530

[7] Crane BT, Tian JR and Demer JL. Initial vestibulo-ocular reflex during transient angular and linear acceleration in human cerebellar dysfunction. Experimental brain research 2000: 130:486-96. doi 10.1007/s002219900266

[8] Dakin CJ, Peters A, Giunti P and Day BL. Cerebellar Degeneration Increases Visual Influence on Dynamic Estimates of Verticality. Current biology : CB 2018: 28:3589-98.e3. doi 10.1016/j.cub.2018.09.049

[9] Gomez CM, Thompson RM, Gammack JT, Perlman SL, Dobyns WB, Truwit CL, Zee DS, Clark HB and Anderson JH. Spinocerebellar ataxia type 6: gaze-evoked and vertical nystagmus, Purkinje cell degeneration, and variable age of onset. Annals of neurology 1997: 42:933-50. doi 10.1002/ana.410420616

[10] Hashimoto T, Sasaki O, Yoshida K, Takei Y and Ikeda S. Periodic alternating nystagmus and rebound nystagmus in spinocerebellar ataxia type 6. Movement disorders : official journal of the Movement Disorder Society 2003: 18:1201-4. doi 10.1002/mds.10511

[11] Huh YE, Kim JS, Kim HJ, Park SH, Jeon BS, Kim JM, Cho JW and Zee DS. Vestibular Performance During High-Acceleration Stimuli Correlates with Clinical Decline in SCA6. Cerebellum (London, England) 2015: 14:284-91. doi 10.1007/s12311-015-0650-3

[12] Inomata-Terada S, Fukuda H, Tokushige SI, Matsuda SI, Hamada M, Ugawa Y, Tsuji S and Terao Y. Abnormal saccade profiles in hereditary spinocerebellar degeneration reveal cerebellar contribution to visually guided saccades. Clinical neurophysiology : official journal of the International Federation of Clinical Neurophysiology 2023: 154:70-84. doi 10.1016/j.clinph.2023.07.006

[13] Joiner WM, Shelhamer M and Ying SH. Cerebellar influence in oculomotor phase-transition behavior. Annals of the New York Academy of Sciences 2005: 1039:536-9. doi 10.1196/annals.1325.062

[14] Kerber KA, Jen JC, Perlman S and Baloh RW. Late-onset pure cerebellar ataxia: differentiating those with and without identifiable mutations. Journal of the neurological sciences 2005: 238:41-5. doi 10.1016/j.jns.2005.06.006

[15] Kim JS, Kim JS, Youn J, Seo DW, Jeong Y, Kang JH, Park JH and Cho JW. Ocular motor characteristics of different subtypes of spinocerebellar ataxia: distinguishing features. Movement disorders : official journal of the Movement Disorder Society 2013: 28:1271-7. doi 10.1002/mds.25464

[16] Kim JM, Nam TS, Choi SM, Kim BC and Lee SH. Clinical value of vestibulo-ocular reflex in the differentiation of spinocerebellar ataxias. Scientific reports 2023: 13:14783. doi 10.1038/s41598-023-41924-6

[17] Lasker AG, Isotalo EH and Zee DS. Predictive saccades to a regularly alternating target in cerebellar patients. Annals of the New York Academy of Sciences 2005: 1039:544-7. doi 10.1196/annals.1325.064

[18] Lee SU, Kim JS, Kim HJ, Choi JY, Park JY, Kim JM and Yang X. Evolution of the vestibular function during head impulses in spinocerebellar ataxia type 6. Journal of neurology 2020: 267:1672-8. doi 10.1007/s00415-020-09756-w

[19] Matsuda S, Matsumoto H, Furubayashi T, Fukuda H, Emoto M, Hanajima R, Tsuji S, Ugawa Y and Terao Y. Top-down but not bottom-up visual scanning is affected in hereditary pure cerebellar ataxia. PloS one 2014: 9:e116181. doi 10.1371/journal.pone.0116181

[20] Matsuda S, Matsumoto H, Furubayashi T, Fukuda H, Hanajima R, Tsuji S, Ugawa Y and Terao Y. Visual scanning area is abnormally enlarged in hereditary pure cerebellar ataxia. Cerebellum (London, England) 2015: 14:63-71. doi 10.1007/s12311-014-0600-5

[21] Takeichi N, Fukushima K, Sasaki H, Yabe I, Tashiro K and Inuyama Y. Dissociation of smooth pursuit and vestibulo-ocular reflex cancellation in SCA-6. Neurology 2000: 54:860-6. doi 10.1212/wnl.54.4.860

[22] Terao Y, Fukuda H, Tokushige S, Inomata-Terada S, Yugeta A, Hamada M, Ichikawa Y, Hanajima R and Ugawa Y. Is multiple system atrophy with cerebellar ataxia (MSA-C) like spinocerebellar ataxia and multiple system atrophy with parkinsonism (MSA-P) like Parkinson's disease? - A saccade study on pathophysiology. Clinical neurophysiology : official journal of the International Federation of Clinical Neurophysiology 2016: 127:1491-502. doi 10.1016/j.clinph.2015.07.035

[23] Terao Y, Fukuda H, Tokushige SI, Inomata-Terada S, Yugeta A, Hamada M and Ugawa Y. Distinguishing spinocerebellar ataxia with pure cerebellar manifestation from multiple system atrophy (MSA-C) through saccade profiles. Clinical neurophysiology : official journal of the International Federation of Clinical Neurophysiology 2017: 128:31-43. doi 10.1016/j.clinph.2016.10.012

[24] Wiest G, Tian JR, Baloh RW, Crane BT and Demer JL. Otolith function in cerebellar ataxia due to mutations in the calcium channel gene CACNA1A. Brain : a journal of neurology 2001: 124:2407-16. doi 10.1093/brain/124.12.2407
